# Role of neutropenic diet in prevention of infection and graft-versus-host disease in haematopoietic stem cell transplant recipients: systematic review and meta-analysis protocol

**DOI:** 10.3389/fnut.2026.1820858

**Published:** 2026-05-26

**Authors:** Manideepa Maji, Saikat Mandal, Arkadeep Dhali, Laura J. Miller, Jane Grove, John A. Snowden, Suparno Chakrabarti, Guruprasad Aithal

**Affiliations:** 1 Hull York Medical School, University of Hull, Kingston upon Hull, United Kingdom; 2 Hull University Teaching Hospitals NHS Trust, Kingston upon Hull, United Kingdom; 3 Nottingham Digestive Disease Centre, Translational Medical Sciences, School of Medicine, University of Nottingham, England, United Kingdom; 4 NIHR Nottingham Biomedical Research Centre, Nottingham University Hospitals NHS Trust and the University of Nottingham, Nottingham, United Kingdom; 5 University of Sheffield, Sheffield, United Kingdom; 6 University of Edinburgh, Edinburgh, United Kingdom; 7 Nottingham University Hospitals NHS Trust, Nottingham, United Kingdom; 8 Division of Clinical Medicine, School of Medicine and Population Health, The University of Sheffield, Sheffield, United Kingdom; 9 Sheffield Teaching Hospitals NHS Foundation Trust, Sheffield, United Kingdom; 10 Action Institute for Blood Diseases, Transplantation & Cellular Therapy (AIBTraCT), Action Cancer Hospital, New Delhi, India; 11 Amity Institute of Mol Med and Stem Cell Research, Amity University, Noida, India; 12 Department of Translational & Clinical Research, Jamia Hamdard University, New Delhi, India

**Keywords:** bone marrow transplant (BMT), diet in bone marrow transplant, graft vs. host disease, haematopoietic stem cell transplant, liberal diet, neutropenic diet

## Abstract

**Introduction:**

Haematopoietic stem cell transplantation (HSCT) is associated with substantial early infectious risk, neutropenia, immunosuppression, and graft-versus-host disease (GVHD). Many centres continue to use neutropenic, low-microbial, low-bacterial, or protective diets to reduce dietary exposure to potential pathogens, despite variation in diet definitions and concerns regarding nutritional intake, patient experience, cost, and microbiota recovery. This protocol describes a systematic review and meta-analysis evaluating the benefits and harms of neutropenic diets compared with less restrictive, standard, or food-safety-based dietary approaches in HSCT recipients.

**Methods:**

We will include randomised and non-randomised comparative studies involving children or adults undergoing HSCT from any graft source and conditioning intensity. Eligible interventions will include neutropenic, low-microbial, low-bacterial, or protective diets; comparators will include unrestricted, less restrictive, standard hospital, or food-safety-based diets. MEDLINE, Embase, CENTRAL, Web of Science, CINAHL, Scopus, ClinicalTrials.gov, and the WHO ICTRP will be searched from inception without language or date restrictions, supplemented by reference screening, expert contact, grey literature, and conference proceedings. Two reviewers will independently screen studies, extract data, and assess risk of bias using RoB 2 for randomised trials and RoBANS for non-randomised studies.

**Results:**

The primary outcomes will be infection rates, acute GVHD, nutritional status, time to neutrophil recovery, and patient satisfaction or quality of life. Secondary outcomes will include overall survival, relapse, chronic GVHD, length of hospitalisation, antibiotic use to day 100, micronutrient deficiency, and cost outcomes. Where appropriate, pooled estimates will be generated using random-effects models, with subgroup and sensitivity analyses used to explore heterogeneity. Certainty of evidence will be assessed using GRADE.

**Discussion:**

This review will clarify whether neutropenic diets reduce infectious complications or GVHD after HSCT, and whether any potential benefit is offset by nutritional, patient-centred, microbiome-related, or economic harms. Findings will inform clinical practice, patient counselling, and future policy on dietary restrictions after HSCT.

**Systematic review registration:**

https://www.crd.york.ac.uk/PROSPERO/view/CRD420251162724, identifier PROSPERO (CRD420251162724).

## Background

Haematopoietic stem cell transplantation (HSCT) represents one of the most important therapeutic modalities for adults with haematological malignancies and selected non-malignant conditions such as aplastic anaemia and haemoglobinopathies (1). By replacing diseased or dysfunctional haematopoietic and immune systems with healthy donor cells, HSCT offers a potential cure. Despite these curative benefits, there are significant mortality and morbidity following HSCT. The major causes of morbidity and mortality following HSCT may be due to the underlying disease and its relapse or related to HSCT, such as graft-versus-host disease (GVHD) specifically for allogenic HSCT recipients, organ damage, and infection (2). Serious complications such as bacterial bloodstream infection have been commonly noted in the early stage of allo-HSCT, with a reported incidence of 13.6–38.9% (3), and infection remains an important cause of non-relapse related death in HSCT patients; during the early phase, infection-related mortality can reach even up to 30% (4).

The intensity and complexity of pre-transplant chemotherapy or radiotherapy conditioning regimens, which aim to ensure immune ablation for engraftment and reduce malignant burden, induce profound immunosuppression (5). This renders recipients highly susceptible to bacterial, viral, and fungal infections, particularly during the early post-transplant phase when neutropenia is most profound. While a strong donor-derived immune response is required to mediate the graft-versus-leukaemia (GvL) effect in malignant indications (6), the same immune reactivity can provoke GvHD, in which donor lymphocytes attack host organs, including the skin, liver, and gastrointestinal tract (7). Conversely, patients undergoing transplantation for benign conditions (such as aplastic anaemia) do not require intensive GvL activity and are often maintained on prolonged immunosuppressive therapy, raising their susceptibility to infections and further complicating post-transplant care.

Infection is not only a direct cause of morbidity and mortality but also contributes to the pathogenesis and exacerbation of GVHD (8, 9). Bacterial infections may stimulate systemic pro-inflammatory cytokine responses that can precipitate or worsen acute GVHD (10, 11). Fungal infections, especially those involving the gastrointestinal (GI) tract, are also recognised as risk factors for refractory or more severe GI-GVHD (12). Thus, rigorous infection prevention strategies are a foundation of post-transplant supportive care.

Effective management of infection and its prevention during the initial phase following HSCT is critical for minimising infection-related mortality and reducing the risk of GVHD. Nutritional management is regarded as an essential aspect of primary prevention strategies during the early post-HSCT period (13). It serves as a foundational supportive measure at this stage, particularly given that malnutrition itself predisposes to infection, impairs wound healing, and hinders immune recovery. Among infection prevention strategies, the neutropenic diet (ND), which is sometimes termed the low-bacterial or low-microbial diet, has been widely implemented in transplant centres worldwide to reduce dietary microbial exposure during neutropenia (14).

The concept underpinning the ND relies on minimising exposure to potential pathogens by restricting or excluding raw fruits, vegetables, unpasteurised dairy, and undercooked animal-source foods. Its widespread adoption originated from theoretical grounds, and at the time of implementation, this approach was not supported by clinical trial evidence (15, 16). Across centres, substantial variation exists in both the definition and enforcement of these dietary restrictions, with some protocols highly restrictive and others more liberal, reflecting the lack of a global standard.

A recent systematic review of cancer patients, which also includes HSCT recipients, examined studies that practised a neutropenic diet and failed to demonstrate (17) that ND alone or in any variant meaningfully reduces infection rates, mortality, or length of hospitalisation following HSCT. The systematic review further determined that the analysed publications suggest a heightened risk of malnutrition, as individuals adhering to an ND more commonly report adverse effects on their nutritional habits. However, no meta-analysis was noted to direct the exact magnitude of the problems for HSCT patients.

A recent systematic review of neutropenic diets in adults with cancer failed to demonstrate (17) that ND alone, or in any variant, meaningfully reduces infection rates, mortality, or length of hospitalisation following HSCT. The systematic review also suggested an increased risk of malnutrition in individuals adhering to an ND (17). However, to date, no meta-analysis has quantified the precise degree of risk reduction for infection, graft-versus-host disease (GvHD), or mortality associated with the use of ND in allo-HSCT recipients.

Among comparable groups of patients with acute myeloid leukaemia undergoing chemotherapy, those who received ND exhibited lower albumin levels than the group that was provided a liberal diet (18, 19). Additionally, some studies have paradoxically reported higher infection rates in recipients adhering to ND regimens, and further reports poorer nutritional intake and negative impacts on patient wellbeing among ND recipients when palatable or familiar foods are excluded. The resulting risk of caloric deficit and micronutrient insufficiency is of real concern, given the high metabolic demands of HSCT recipients (20, 21). Furthermore, current European Society for Blood and Marrow Transplantation (EBMT) and European Society for Clinical Nutrition and Metabolism (ESPEN) supportive care guidelines have moved away from endorsing the ND, instead recommending adherence to meticulous food safety and hygiene principles (22, 23), while American health organizations have already issued recommendations on replacing the ND with safe food-handling guidelines (24, 25). Nonetheless, a significant number of transplant centres (86%) continue ND practices rooted in tradition or local policies, reflecting the inertia of long-standing clinical practice and, in certain contexts, cultural attitudes toward infection risk (14). On the contrary, fibre-rich fruits and vegetables can serve as substrates for short-chain fatty acid production; these fatty acids have been observed to support immune reconstitution and may suppress the development of GvHD after allogenic HSCT (allo-HSCT) (26, 27). Recent research also suggests ND delays the recovery of gut microbiota diversity. Problematically, during HSCT, patients receive high-dose chemotherapy at the time of conditioning, which can cause gut dysbiosis—loss of diversity or changes to the gut microbiota and a prolonged neutropenic diet further delays the recovery. Together, these have the potential to affect neutrophil recovery and eventually to increase the risk of infection and GvHD (28–30). The implementation of neutropenic diets and related low-microbial dietary interventions imposes additional costs on healthcare systems or on patients’ out-of-pocket expenses, particularly in insurance-based settings.

Given the ongoing controversies, the lack of a unified standard of care, the increased cost burden, and the suboptimal patient experience associated with ND-related practices, a systematic review and meta-analysis of ND and related low-microbial diets in the context of HSCT is urgently needed. Synthesising the available clinical trials (31, 32) and observational evidence (20, 33, 34) can clarify the true magnitude of benefit or harm associated with ND, and directly inform clinical practice guidelines, patient counselling, and institutional protocols. Key clinical outcomes to be evaluated include the incidence of infections (bacterial, fungal, and viral), the occurrence and severity of acute or chronic GvHD among allogenic HSCT recipients, the length of hospitalisation, overall survival, relapse risk, and nutritional status, including its impact on malnutrition risk. The review should also explore variation in ND definitions, duration, and how these relate to patient outcomes, as well as patient-reported experiences of diet-related quality of life.

Having conducted pilot work in this area, the authors now aim to undertake a comprehensive systematic review and meta-analysis to accurately characterise the impact of neutropenic, low-microbial, or low-bacterial diets compared with liberal dietary approaches in HSCT recipients, irrespective of whether dietitians are engaged in the management plan. This will encompass studies of diverse methodology, including randomised and non-randomised designs, and will focus in particular on the early post-transplant period when infection risk is highest and dietary interventions are most commonly implemented.

### Review objectives

To conduct a comprehensive systematic review and meta-analysis evaluating the impact of neutropenic, low-bacterial, or low-microbial diets on clinical outcomes in recipients of haematopoietic stem cell transplantation (HSCT) during the early post-transplant period.

## Methods

### Population

The population considered in this review includes all age group, i.e., both children and adults undergoing HSCT. These individuals may receive grafts from various sources, such as bone marrow, peripheral blood, or cord blood, and undergo a range of conditioning regimens prior to transplantation.

### Interventions

#### Type of intervention

Restrictive dietary strategy (neutropenic/protective/low-microbial/low bacterial diet) minimising ingestion of potential pathogens via restriction or exclusion of raw fruits, vegetables, unpasteurised dairy, and undercooked animal-source foods.

### Comparison

These interventions are compared against all unrestricted diets, less restrictive diets (in terms of infection risk), diets according to safe food handling recommendations and any normal hospital diet.

### Outcome measures

The following outcome measures will be used to evaluate the effectiveness and safety of the interventions under consideration:

#### Primary outcomes

- *Infection rates:* incidences of bacterial, viral, and fungal infections reported in the individual included studies will be tracked as part of the dietary intervention’s efficacy assessment.
- *Incidence of acute graft-versus-host disease (GVHD)* affecting the gastrointestinal tract, liver and Skin will be checked for allogenic HSCT recipients. This will be assessed for grades II–IV as reported in the included studies.
- *Nutritional parameters:* the review will report changes in participants’ nutritional status, including body composition (body fat mass/muscle mass), body mass index (BMI), malnutrition (validated measure or definition), and changes in weight and dietary intake. These indicators will be used to evaluate the impact of ND on the nutritional status of the participants.
- *Absolute neutrophil count (ANC):* for the purposes of this review, an ANC greater than 0.5 ×10^9/L will be considered as an important parameter for immune reconstitution and engraftment. The onset at which ANC > 500 reached will be considered in evaluating the efficacy of the dietary interventions.
- *Patient satisfaction, acceptability and quality of life (QoL):* measures of satisfaction, acceptability of the ND or low-microbial dietary approaches and overall quality of life related data will be extracted and compared to assess the broader effects of the dietary strategies on patient well-being.

### Secondary outcome

- *Overall survival:* the overall survival rate of patients in the intervention and comparator groups will be documented.
- *Relapse rate*: The number of patients who had a relapse of the underlying disease in the intervention and comparator groups will be documented.
- *Incidence of chronic graft-versus-host disease:* chronic GVHD and its severity will be measured as defined in the study for the allogenic HSCT recipients.
- Length of hospitalisation.
- Empirical antibiotic usage till day 100 of transplant.
- Micronutrient deficiency.
- *Cost economic analysis*: cost burden related to ND approaches will be analysed.

### Types of studies

This review will consider specific types of studies for inclusion and exclusion based on their design and methodological rigour.

#### Inclusion criteria

Studies eligible for inclusion employ robust comparative methodologies. This includes:

- Randomised controlled trials (RCTs), which provide high-quality evidence by randomly assigning participants to intervention or control groups. Cluster-RCTs and quasi-experimental designs will also be considered.
- Non-randomised comparative studies, such as prospective or retrospective cohort studies that include a comparator group. These studies contribute valuable insights by comparing outcomes between groups exposed to different interventions or exposures.

#### Exclusion criteria

The following types of studies will be excluded from the review:

- Case reports and case series, as they lack comparator groups and are generally considered to provide lower levels of evidence.
- Single-arm studies without a comparator, which do not allow for direct comparison between interventions.
- Review articles, editorials, and animal studies, since these do not provide original data from human participants with comparative outcomes.

### Search methods for identification of studies

#### Electronic searches

A comprehensive literature search will be undertaken across multiple electronic databases to ensure the identification of all relevant studies from their inception to the present. The databases to be searched include:

- *MEDLINE (accessible via PubMed or Ovid):* This database will be searched to capture biomedical literature, including clinical research related to haematopoietic stem cell transplantation and associated interventions.
- *Embase:* Embase will be included to retrieve studies indexed in this biomedical and pharmacological database, ensuring comprehensive coverage of international literature.
- *Cochrane Central Register of Controlled Trials (CENTRAL):* CENTRAL will be searched to identify randomised and controlled clinical trials relevant to the research topic.
- *Web of Science:* This multidisciplinary database will be utilised to capture a broad spectrum of scientific research articles and conference proceedings.
- *CINAHL:* The Cumulative Index to Nursing and Allied Health Literature (CINAHL) will be searched for nursing and allied health literature pertinent to the review.
-
*ClinicalTrials.gov* *and WHO International Clinical Trials Registry Platform (ICTRP):* These platforms will be searched to identify ongoing and completed clinical trials, including those that may not yet have been published in peer-reviewed journals.
- *Scopus:* Scopus will be searched from inception to identify additional biomedical and multidisciplinary publications (including conference abstracts) relevant to HSCT and neutropenic/low-microbial dietary interventions

This multi-database approach is intended to maximise the retrieval of eligible studies and minimise publication bias, supporting a comprehensive and systematic review process.

#### Search strategy

- Search terms will include combinations of controlled vocabulary (MeSH, Emtree) and keywords for “hematopoietic stem cell transplantation”, AND “neutropenic diet”, OR “restrictive diet”, OR “low-microbial diet”, OR “low-bacterial Diet”, OR “protective diet”, OR “germ-free diet”, OR “no microbial diet”, OR “sterilised diet”, OR “clean diet” OR “food safety.”
- No language, publication status, or date restrictions will be applied.
- The search strategy will be developed in consultation with an information specialist and peer-reviewed in accordance with PRESS guidelines.
- The full search strategy for each database will be provided in an Appendix.
- Please check the detailed search strategy in Appendix 1.

#### Searching other resources

In addition to electronic database searches, several supplementary strategies will be employed to ensure a comprehensive identification of relevant studies:

- Hand-searching Reference Lists: The reference lists of all included studies and pertinent review articles will be meticulously hand-searched to identify additional studies that may not have been captured through database searches.
- Contacting Experts and Authors: Subject-matter experts and corresponding authors of included studies will be contacted to enquire about any ongoing or unpublished studies that are relevant to the review topic at least twice, with an interval of 7 days before declaring non-responder.
- Review of Conference Proceedings: Proceedings from major conferences, such as those organised by the British Association of Parenteral and Enteral Nutrition (BAPEN), Academy of Nutrition and Dietetics, The European Federation of the Associations of Dietitians (EFAD), American Society of Hematology (ASH) and the European Society for Blood and Marrow Transplantation (EBMT), from the past 5 years will be examined to identify relevant abstracts and presentations.
- Grey Literature Search: Regulatory agency websites and thesis or dissertation databases, such as ProQuest, will be searched to capture additional grey literature that may not be indexed in standard bibliographic databases.

### Data collection and analysis

#### Selection of studies

The process of selecting studies for inclusion in this review will be conducted by two independent reviewers. Initially, both reviewers will screen the titles and abstracts of all identified records to determine eligibility using the predefined inclusion and exclusion criteria. For studies that appear potentially relevant, the full-text articles will be retrieved and independently assessed by both reviewers to confirm eligibility.

In cases where there is disagreement between the reviewers regarding the eligibility of a study, the issue will be resolved through discussion. If consensus cannot be reached, a third reviewer will be consulted to provide a final decision.

The study selection process will be thoroughly documented using a PRISMA (Preferred Reporting Items for Systematic Reviews and Meta-Analyses) flow diagram (35). This diagram will detail the number of records identified, screened, excluded, and included at each stage, and will also specify the reasons for exclusion at the full-text screening stage.

#### Data extraction and management

- Data will be extracted independently by two reviewers using a standardised, piloted data extraction form.
- Extracted data will include:
  - Study identification (author, year, journal, country, funding)
  - Study design and setting
  - Participant characteristics (age, sex, underlying disease, conditioning regimen, graft source)
  - Intervention details (type, duration, onset)
  - Comparator details
  - Outcomes (definitions, time points, effect estimates, confidence intervals, *p*-values)
  - Risk of bias-related information (randomisation, blinding, attrition, confounding)
  - Other relevant findings (subgroup analyses, limitations, deviations from protocol)
- Discrepancies will be resolved by consensus or third-party adjudication.
- Data will be managed using Review Manager (Covidence) or equivalent software.

### Assessment of risk of bias

#### Randomised controlled trials (RCTs)

The risk of bias in randomised controlled trials will be evaluated using the Cochrane Risk of Bias 2 (RoB 2) tool (36). This tool systematically examines several domains, including the process of randomisation, deviations from intended interventions, handling of missing data, outcome measurement, and the potential for selective reporting. Each domain will be assessed to determine the overall risk of bias for each included RCT.

#### Non-randomised studies

For non-randomised studies, the risk of bias will be assessed using the risk of bias assessment tool for non-randomised studies (RoBANS) (37). RoBANS assesses similar domains adapted for observational research (e.g., selection of participants, confounding variables, measurement of exposure/intervention, blinding of outcome assessment, incomplete outcome data, and selective outcome reporting). Again, each domain will be judged as low, high, or unclear risk of bias.

#### Review process

Two reviewers will independently assess the risk of bias for all included studies. In the event of disagreements, these will be resolved through discussion. If consensus cannot be reached, a third reviewer will adjudicate to ensure objectivity and accuracy in the assessment.

#### Presentation of results

The results of the risk of bias assessments will be summarised in tables. These summaries will be incorporated into the interpretation of findings to contextualise the strengths and limitations of the available evidence.

#### Measures of treatment effect

- *Dichotomous outcomes:* (e.g., mortality, occurrence of infection events)
  - Risk ratios (RRs) or odds ratios (ORs) with 95% confidence intervals (CIs).
- *Continuous outcomes:* (e.g., quality of life scores)
  - Mean differences (MDs) or standardised mean differences (SMDs) with 95% CIs.
- *Time-to-event outcomes:*
  - Incidence of GVHD: cumulative incidence estimates accounting for competing risks (e.g., death without GVHD) will be preferred where reported. Hazard ratios (HRs) with 95% CIs will be extracted or approximated from survival data. Where only dichotomous GVHD data are available, these will be analysed using risk ratios.
- *Absolute effects:*
  - Risk differences and number needed to treat (NNT) or harm (NNH) will be calculated where possible.

#### Unit of analysis issues

We anticipate that the unit of analysis will be the individual patient in all included studies. Cluster-randomised trials or cross-over trial designs are unlikely in this context (it is improbable to cluster by centre or to cross patients over between transfusion policies). If we do encounter a cluster-randomised trial, we will ensure appropriate analysis by checking that clustering has been accounted for in the published analysis, or by adjusting the effective sample size using intra-cluster correlation coefficients if necessary, according to Cochrane Handbook guidance (38). For any cross-over trials (also unexpected in this context), we would use data from the first phase only (to avoid carry-over effects) or otherwise treat them cautiously as outlined in the Handbook. If multiple observations per participant are reported (e.g., repeated measurements over time), we will either choose a consistent time point for analysis or use methods for longitudinal data if meta-analysis is feasible, ensuring that each participant’s data are analysed only once for each outcome. In summary, any unit-of-analysis issues identified will be handled in accordance with Cochrane recommendations to avoid unit-of-analysis errors (38).

#### Dealing with missing data

In instances where data are missing or unclear, the study authors will be contacted to obtain the necessary information. This approach is intended to ensure the completeness and accuracy of the data used in the analysis.

When feasible, analyses will be undertaken on an intention-to-treat (ITT) basis. This means that all participants originally allocated to each group will be included in the analysis, regardless of whether they completed the intervention as per the study protocol.

For each study, the extent of missing data and the strategies employed to manage it will be carefully documented. This documentation will provide transparency and facilitate understanding of how missing data may influence the results.

Sensitivity analyses will be conducted to evaluate the effect of missing data on the study findings. These analyses will help to determine the robustness of the results under different assumptions about the missing data.

#### Assessment of heterogeneity

To ensure the validity and reliability of the meta-analysis, both clinical and methodological heterogeneity will be systematically assessed by thoroughly examining the characteristics of the included studies. This will involve a detailed comparison of study designs, populations, interventions, and outcomes to identify any notable differences that could influence the results.

Statistical heterogeneity will be formally evaluated using the chi-squared (*χ*^2^) test, with a significance threshold set at *p* < 0.1, and the *I*^2^ statistic. The *I*^2^ value will be interpreted as follows:

- *I*^2^ > 50% will be considered indicative of moderate heterogeneity.
- *I*^2^ > 80% will be regarded as substantial heterogeneity.

In the presence of heterogeneity, potential sources will be explored through subgroup analyses and sensitivity analyses. These methods will help to identify and understand the underlying factors contributing to variability among study findings, thereby strengthening the interpretation and robustness of the overall synthesis.

#### Assessment of reporting biases

If a meta-analysis includes 10 or more studies, publication bias will be formally evaluated by constructing funnel plots to visually inspect for asymmetry. In addition, Egger’s regression test (39) will be performed to statistically assess the presence of publication bias within the included studies.

To identify outcome reporting bias, the study protocols or registrations will be compared with the outcomes reported in the published articles. This comparison will help detect discrepancies and selective reporting that may influence the interpretation of the results.

The potential impact of suspected reporting biases on the findings will be thoroughly discussed, considering how these biases may affect the interpretation and validity of the meta-analysis outcomes.

### Data synthesis

Meta-analyses will be conducted utilising random-effects models, specifically the DerSimonian-Laird (40) method, to account for variability between studies. This approach ensures that differences across studies are appropriately considered, providing more generalisable results.

For dichotomous outcomes, the Mantel–Haenszel (41) method will be applied to calculate pooled effect estimates. In the case of continuous outcomes, the inverse-variance method will be employed to synthesise data. For time-to-event outcomes, pooling will be performed using the generic inverse-variance method, allowing for the integration of hazard ratios or other relevant effect measures.

To assess the robustness of the findings, fixed-effect models will be used in sensitivity analyses. This will help determine whether the choice of statistical model influences the results of the meta-analysis.

In circumstances where meta-analysis is not feasible due to factors such as insufficient data or substantial heterogeneity, a narrative synthesis will be undertaken. Studies will be grouped according to intervention type, outcome, or population, as appropriate, to provide a structured summary of the findings.

We will prepare a Summary of Findings table for the main comparison (infection rate, incidence and severity of GVHD) and key outcomes, following the GRADE approach to assess the certainty of evidence. Outcomes to be included in the Summary of Findings table will likely include quality of life, all-cause mortality, serious transfusion-related adverse events, cardiac events, and transfusion requirements (as these reflect both benefits and harms and are of high importance to decision-makers). We will use GRADEpro software to construct this table and will justify any downgrading or upgrading of evidence quality in footnotes, according to GRADE criteria (risk of bias, inconsistency, indirectness, imprecision, and publication bias).

### Subgroup analysis and investigation of heterogeneity

Where data permit, subgroup analyses will be performed by:

- Study design (RCT vs. non-randomised)
- Conditioning regimen intensity (myeloablative vs. reduced intensity)
- Donor source, i.e., matched sibling donor (MSD), matched unrelated donor (MUD), mismatched unrelated donor (MMUD), and haploidentical donor.
- Graft source, e.g., bone marrow, peripheral blood, cord blood. Cord blood will be analysed as a separate graft-source subgroup only when sufficient data are available for meaningful comparison.
- Age group such as adult and paediatric
- Underlying disease, i.e., benign condition and malignancy
- T-cell depletion strategy [e.g., post-transplant cyclophosphamide (PTCy), anti-thymocyte globulin (ATG)], where reported.
- Geographic location (high-income vs. low- and middle-income countries)

Subgroup differences will be tested using chi-squared tests for interaction. Results will be interpreted cautiously, recognizing the exploratory nature of subgroup analyses.

### Sensitivity analysis

To evaluate the robustness and reliability of the primary findings, a series of sensitivity analyses will be conducted. These analyses are designed to determine whether the main results are influenced by specific methodological or analytical decisions. The following approaches will be implemented:

- Exclusion of studies at high risk of bias: analyses will be repeated after removing studies that are assessed as having a high risk of bias, to examine whether such exclusions materially affect the overall findings.
- Exclusion of studies with high attrition or missing data: studies characterised by high rates of participant loss or substantial missing data will be excluded in additional analyses to assess their impact on the results.
- Restriction to randomised controlled trials (RCTs) only: sensitivity analyses will be performed by including only RCTs, thereby evaluating whether the findings are consistent when limiting to the highest level of evidence.
- Comparison of fixed-effect and random-effects models: the choice of statistical model can influence results; therefore, analyses will be conducted using both fixed-effect and random-effects models to assess the impact on effect estimates.

Any changes to the estimates of effects or conclusions resulting from these sensitivity analyses will be carefully noted and discussed in the review. This process will help ensure that the conclusions drawn are robust and reflective of the underlying evidence, accounting for potential sources of bias or heterogeneity in the included studies.

All methods outlined above align with the Cochrane Handbook for Systematic Reviews of Interventions (38) and PRISMA reporting guidelines (42), thereby ensuring the transparency, reproducibility, and rigour of our systematic review methodology. The review will be conducted and reported in full compliance with established methodological standards, aiming to provide rigorous evidence on the effectiveness of the neutropenic diet in preventing infection and graft-versus-host disease among adult recipients of HSCT.

## Discussion

This review addresses a persistent and practice-relevant uncertainty in HSCT supportive care: despite widespread historical use, neutropenic/low-microbial diets are variably defined and increasingly questioned, with emerging guidance favouring safe food-handling approaches over blanket dietary restriction. However, a recently completed randomised controlled trial still provides evidence supporting the continuation of a neutropenic diet after hematopoietic stem cell transplant (43). By synthesising comparative evidence across randomised and non-randomised designs, we aim to quantify whether restrictive diets meaningfully reduce infectious complications or GVHD, and whether any potential benefits are offset by harms, including reduced intake, increased risk of malnutrition, impaired patient experience, or delayed microbiota recovery. We anticipate important clinical and methodological heterogeneity (e.g., variation in diet stringency, timing/duration, conditioning intensity, antimicrobial prophylaxis, paediatric vs. adult populations), which will be explored through pre-specified subgroup and sensitivity analyses. The certainty of the evidence will be assessed transparently (risk of bias, inconsistency, imprecision, indirectness, and publication bias), ensuring that conclusions are proportionate to the underlying data. Findings will directly guide patient counselling and service design, and where suitable, support the rational removal of restrictive diets in favour of evidence-based food safety measures that better maintain nutritional status and quality of life.
