# Supplementary material for: Role of neutropenic diet in prevention of infection and graft-versus-host disease in haematopoietic stem cell transplant recipients: systematic review and meta-analysis protocol
Source: Front Nutr. 2026 May 26;13:1820858. doi: 10.3389/fnut.2026.1820858 (PMC13246377; doi:10.3389/fnut.2026.1820858)
Supplement: Supplementary file 1 [file Supplementary_File_1.docx]

**Appendix 1:**

**Database: Embase <1974 to 2025 Week 40>**

**Search Strategy:**

--------------------------------------------------------------------------------

1 exp peripheral blood stem cell transplantation/ or exp bone marrow transplantation/ (82744)

2 Peripheral Blood Stem Cell Transplantation.mp. [mp=title, abstract, heading word, drug trade name, original title, device manufacturer, drug manufacturer, device trade name, keyword heading word, floating subheading word, candidate term word] (10184)

3 Haematopoietic Stem Cell Transplantation.mp. [mp=title, abstract, heading word, drug trade name, original title, device manufacturer, drug manufacturer, device trade name, keyword heading word, floating subheading word, candidate term word] (6017)

4 exp hematopoietic stem cell transplantation/ (103718)

5 exp bone marrow transplantation/ (75801)

6 Bone marrow transplant.mp. [mp=title, abstract, heading word, drug trade name, original title, device manufacturer, drug manufacturer, device trade name, keyword heading word, floating subheading word, candidate term word] (14102)

7 exp hematopoietic stem cell transplantation/ (103718)

8 H?ematopoietic Stem Cell Transplant*.mp. [mp=title, abstract, heading word, drug trade name, original title, device manufacturer, drug manufacturer, device trade name, keyword heading word, floating subheading word, candidate term word] (118218)

9 Bone Marrow Transplant*.mp. [mp=title, abstract, heading word, drug trade name, original title, device manufacturer, drug manufacturer, device trade name, keyword heading word, floating subheading word, candidate term word] (85003)

10 Peripheral Blood Stem Cell Transplant*.mp. [mp=title, abstract, heading word, drug trade name, original title, device manufacturer, drug manufacturer, device trade name, keyword heading word, floating subheading word, candidate term word] (10830)

11 neutropenic diet.mp. (125)

12 neutropenic diet.mp. [mp=title, abstract, heading word, drug trade name, original title, device manufacturer, drug manufacturer, device trade name, keyword heading word, floating subheading word, candidate term word] (125)

13 restrictive diet.mp. [mp=title, abstract, heading word, drug trade name, original title, device manufacturer, drug manufacturer, device trade name, keyword heading word, floating subheading word, candidate term word] (631)

14 exp diet restriction/ (188940)

15 low-microbial diet.mp. (24)

16 low*microbial diet.mp. (1)

17 low-bacterial Diet.mp. [mp=title, abstract, heading word, drug trade name, original title, device manufacturer, drug manufacturer, device trade name, keyword heading word, floating subheading word, candidate term word] (39)

18 low*bacterial Diet.mp. [mp=title, abstract, heading word, drug trade name, original title, device manufacturer, drug manufacturer, device trade name, keyword heading word, floating subheading word, candidate term word] (1)

19 protective diet.mp. [mp=title, abstract, heading word, drug trade name, original title, device manufacturer, drug manufacturer, device trade name, keyword heading word, floating subheading word, candidate term word] (85)

20 germ-free diet.mp. (2)

21 germ-free diet.mp. [mp=title, abstract, heading word, drug trade name, original title, device manufacturer, drug manufacturer, device trade name, keyword heading word, floating subheading word, candidate term word] (2)

22 germ*free diet.mp. [mp=title, abstract, heading word, drug trade name, original title, device manufacturer, drug manufacturer, device trade name, keyword heading word, floating subheading word, candidate term word] (2)

23 no microbial diet.mp. [mp=title, abstract, heading word, drug trade name, original title, device manufacturer, drug manufacturer, device trade name, keyword heading word, floating subheading word, candidate term word] (0)

24 no*microbial diet.mp. [mp=title, abstract, heading word, drug trade name, original title, device manufacturer, drug manufacturer, device trade name, keyword heading word, floating subheading word, candidate term word] (1)

25 sterili?ed diet.mp. [mp=title, abstract, heading word, drug trade name, original title, device manufacturer, drug manufacturer, device trade name, keyword heading word, floating subheading word, candidate term word] (9)

26 1 or 2 or 3 or 4 or 5 or 6 or 7 or 8 or 9 or 10 (199268)

27 11 or 12 or 13 or 14 or 15 or 16 or 17 or 18 or 19 or 20 or 21 or 22 or 23 or 24 or 25 (189544)

28 26 and 27 (331)

**Database: Ovid MEDLINE(R) ALL <1946 to October 02, 2025>**

**Search Strategy:**

--------------------------------------------------------------------------------

1 exp Hematopoietic Stem Cell Transplantation/ or exp Graft vs Host Disease/ (78490)

2 exp Hematopoietic Stem Cell Transplantation/ (62996)

3 exp Hematopoietic Stem Cell Transplantation/ or exp Graft vs Host Disease/ or Haematopoietic Stem Cell Transplantation.mp. (79469)

4 Hematopoietic Stem Cell Transplantation.mp. [mp=title, book title, abstract, original title, name of substance word, subject heading word, floating sub-heading word, keyword heading word, organism supplementary concept word, protocol supplementary concept word, rare disease supplementary concept word, unique identifier, synonyms, population supplementary concept word, anatomy supplementary concept word] (69707)

5 Haematopoietic Stem Cell Transplantation.mp. [mp=title, book title, abstract, original title, name of substance word, subject heading word, floating sub-heading word, keyword heading word, organism supplementary concept word, protocol supplementary concept word, rare disease supplementary concept word, unique identifier, synonyms, population supplementary concept word, anatomy supplementary concept word] (3191)

6 exp Peripheral Blood Stem Cell Transplantation/ or exp Bone Marrow Transplantation/ or exp Hematopoietic Stem Cell Transplantation/ (105100)

7 Peripheral Blood Stem Cell Transplantation.mp. [mp=title, book title, abstract, original title, name of substance word, subject heading word, floating sub-heading word, keyword heading word, organism supplementary concept word, protocol supplementary concept word, rare disease supplementary concept word, unique identifier, synonyms, population supplementary concept word, anatomy supplementary concept word] (5916)

8 H?ematopoietic Stem Cell Transplant*.mp. [mp=title, book title, abstract, original title, name of substance word, subject heading word, floating sub-heading word, keyword heading word, organism supplementary concept word, protocol supplementary concept word, rare disease supplementary concept word, unique identifier, synonyms, population supplementary concept word, anatomy supplementary concept word] (73032)

9 Bone Marrow Transplant*.mp. [mp=title, book title, abstract, original title, name of substance word, subject heading word, floating sub-heading word, keyword heading word, organism supplementary concept word, protocol supplementary concept word, rare disease supplementary concept word, unique identifier, synonyms, population supplementary concept word, anatomy supplementary concept word] (58995)

10 exp Bone Marrow Transplantation/ (46110)

11 neutropenic diet.mp. (68)

12 neutropenic diet.mp. [mp=title, book title, abstract, original title, name of substance word, subject heading word, floating sub-heading word, keyword heading word, organism supplementary concept word, protocol supplementary concept word, rare disease supplementary concept word, unique identifier, synonyms, population supplementary concept word, anatomy supplementary concept word] (68)

13 restrictive diet.mp. [mp=title, book title, abstract, original title, name of substance word, subject heading word, floating sub-heading word, keyword heading word, organism supplementary concept word, protocol supplementary concept word, rare disease supplementary concept word, unique identifier, synonyms, population supplementary concept word, anatomy supplementary concept word] (315)

14 restrictive diet.mp. (315)

15 low-microbial diet.mp. (12)

16 low*microbial diet.mp. [mp=title, book title, abstract, original title, name of substance word, subject heading word, floating sub-heading word, keyword heading word, organism supplementary concept word, protocol supplementary concept word, rare disease supplementary concept word, unique identifier, synonyms, population supplementary concept word, anatomy supplementary concept word] (0)

17 low-bacterial Diet.mp. [mp=title, book title, abstract, original title, name of substance word, subject heading word, floating sub-heading word, keyword heading word, organism supplementary concept word, protocol supplementary concept word, rare disease supplementary concept word, unique identifier, synonyms, population supplementary concept word, anatomy supplementary concept word] (16)

18 low-bacterial Diet.mp. (16)

19 low*bacterial Diet.mp. [mp=title, book title, abstract, original title, name of substance word, subject heading word, floating sub-heading word, keyword heading word, organism supplementary concept word, protocol supplementary concept word, rare disease supplementary concept word, unique identifier, synonyms, population supplementary concept word, anatomy supplementary concept word] (0)

20 protective diet.mp. [mp=title, book title, abstract, original title, name of substance word, subject heading word, floating sub-heading word, keyword heading word, organism supplementary concept word, protocol supplementary concept word, rare disease supplementary concept word, unique identifier, synonyms, population supplementary concept word, anatomy supplementary concept word] (58)

21 protective diet.mp. (58)

22 germ free diet.mp. (2)

23 germ-free diet.mp. (2)

24 germ-free diet.mp. [mp=title, book title, abstract, original title, name of substance word, subject heading word, floating sub-heading word, keyword heading word, organism supplementary concept word, protocol supplementary concept word, rare disease supplementary concept word, unique identifier, synonyms, population supplementary concept word, anatomy supplementary concept word] (2)

25 germ*free diet.mp. [mp=title, book title, abstract, original title, name of substance word, subject heading word, floating sub-heading word, keyword heading word, organism supplementary concept word, protocol supplementary concept word, rare disease supplementary concept word, unique identifier, synonyms, population supplementary concept word, anatomy supplementary concept word] (0)

26 no microbial diet.mp. [mp=title, book title, abstract, original title, name of substance word, subject heading word, floating sub-heading word, keyword heading word, organism supplementary concept word, protocol supplementary concept word, rare disease supplementary concept word, unique identifier, synonyms, population supplementary concept word, anatomy supplementary concept word] (0)

27 no*microbial diet.mp. [mp=title, book title, abstract, original title, name of substance word, subject heading word, floating sub-heading word, keyword heading word, organism supplementary concept word, protocol supplementary concept word, rare disease supplementary concept word, unique identifier, synonyms, population supplementary concept word, anatomy supplementary concept word] (1)

28 sterili?ed diet.mp. [mp=title, book title, abstract, original title, name of substance word, subject heading word, floating sub-heading word, keyword heading word, organism supplementary concept word, protocol supplementary concept word, rare disease supplementary concept word, unique identifier, synonyms, population supplementary concept word, anatomy supplementary concept word] (11)

29 1 or 2 or 3 or 4 or 5 or 6 or 7 or 8 or 9 or 10 (135685)

30 11 or 12 or 13 or 14 or 15 or 16 or 17 or 18 or 19 or 20 or 21 or 22 or 23 or 24 or 25 or 26 or 27 or 28 (471)

31 29 and 30 (28)
